# Patient-specific positioning guides for total knee arthroplasty: no significant difference between final component alignment and pre-operative digital plan except for tibial rotation

**DOI:** 10.1007/s00167-015-3661-1

**Published:** 2015-06-09

**Authors:** Bert Boonen, Martijn G. M. Schotanus, Bart Kerens, Frans-Jan Hulsmans, Wim E. Tuinebreijer, Nanne P. Kort

**Affiliations:** 1 Department of Orthopaedics, P. Debyelaan 25, 6229 HX Maastricht, The Netherlands; 2 Department of Orthopaedics, Orbis Medical Centre, Dr. H. van der Hoffplein 1, 6162 BG Sittard-Geleen, The Netherlands; 3 Department of Orthopaedics, AZ Sint-Maarten, Leopoldstraat 2, 2800 Mechelen, Belgium; 4 Department of Radiology, Orbis Medical Centre, Dr. H. van der Hoffplein 1, 6162 BG Sittard-Geleen, The Netherlands; 5 Department of Surgery-Traumatology, Erasmus MC, ‘s-Gravendijkwal 230, 3015 CE Rotterdam, The Netherlands

**Keywords:** Total knee arthroplasty, Patient-specific positioning guides, Accuracy, Alignment, 3D CT scan

## Abstract

**Purpose:**

To assess whether there is a significant difference between the alignment of the individual femoral and tibial components (in the frontal, sagittal and horizontal planes) as calculated pre-operatively (digital plan) and the actually achieved alignment in vivo obtained with the use of patient-specific positioning guides (PSPGs) for TKA. It was hypothesised that there would be no difference between post-op implant position and pre-op digital plan.

**Methods:**

Twenty-six patients were included in this non-inferiority trial. Software permitted matching of the pre-operative MRI scan (and therefore calculated prosthesis position) to a pre-operative CT scan and then to a post-operative full-leg CT scan to determine deviations from pre-op planning in all three anatomical planes.

**Results:**

For the femoral component, mean absolute deviations from planning were 1.8° (SD 1.3), 2.5° (SD 1.6) and 1.6° (SD 1.4) in the frontal, sagittal and transverse planes, respectively. For the tibial component, mean absolute deviations from planning were 1.7° (SD 1.2), 1.7° (SD 1.5) and 3.2° (SD 3.6) in the frontal, sagittal and transverse planes, respectively. Absolute mean deviation from planned mechanical axis was 1.9°. The a priori specified null hypothesis for equivalence testing: the difference from planning is >3 or <−3 was rejected for all comparisons except for the tibial transverse plane.

**Conclusion:**

PSPG was able to adequately reproduce the pre-op plan in all planes, except for the tibial rotation in the transverse plane. Possible explanations for outliers are discussed and highlight the importance for adequate training surgeons before they start using PSPG in their day-by-day practise.

**Level of evidence:**

Prospective cohort study, Level II.

## Introduction

Total knee arthroplasty (TKA) is one of the most successful operative procedures with both excellent short-term survival rates [12] and long-term survival. These rates vary between 91 and 95 % for a reported follow-up of 15–23 years [10, 16, 19–21]. Besides the design of the prosthesis, the technical aspects (i.e. surgical skills) are key success elements. Outlining of the prosthesis is one of these essential elements. Although several techniques for outlining of the prosthesis exist nowadays, there is still room for techniques that try to optimise both the efficacy of the operative procedure itself and the accuracy of prosthesis alignment. Moreover, with the growing need for joint replacement surgery, changes will have to be made in multiple areas of the process to meet the future growing demands [11]. A relatively new development to improve alignment and optimise the operative process is the use of patient-specific positioning guides (PSPGs) to determine the appropriate three-dimensional resections of femur and tibia in preparation of prosthesis placement. This technique is either MRI or CT based, meaning that either of these imaging techniques are used to create a pre-operative, 3D image of the individual patient’s knee. These images are subsequently used to calculate ideal implant position using predetermined reference axes and planes. A pre-operative plan is created this way, showing expected implant positioning after surgery. The purpose of PSPG is to create guides that have only one fitting position on the native anatomy of the individual patient. These guides serve as peroperative guiding instruments to place the pins needed to make the bony resections. Most major orthopaedic companies have launched a PSPG system throughout the last years. All use the same basic principles but have specific algorithms.

Theoretically, this method of alignment would help improve implant positioning, would eliminate variability among different surgeons and would optimise the efficacy of surgery. Numerous reports have been published addressing alignment obtained with PSPG. Most recent studies compare the final alignment of the prosthesis with results obtained by conventional instruments using standard radiographs, long-leg radiographs or 2D CT scans. Moreover, these studies use reference axes and reference points on post-operative CT scans to measure alignment of the prostheses components that are not all equal to the reference points and axes used to calculate implant position and subsequently resulting in the pre-operative digital plan (different manufacturers use different calculation algorithms based on different reference axes and points). However, when using such a methodology, potential bias exists and it cannot be determined to what extent the technique is capable of reproducing the pre-operative digital plan that forms the basis of this alignment technique. This step is essential when searching for potential weak spots associated with PSPG, but is not addressed in current literature. To our knowledge, no studies have yet been conducted that directly compared ultimate implant position in all three anatomical planes obtained with PSPG to the pre-operative digital plan that should have dictated this post-operative alignment using a reliable 3D technique.

This study was designed to address the following research question: to assess whether there is a significant difference between the alignment of the individual femoral and tibial components (in the frontal, sagittal and horizontal planes) as calculated pre-operatively (digital plan) and the actually achieved alignment in vivo of an experienced PSPG user. It was hypothesised that there would be no significant difference between the pre-operative alignment as determined by software and the ultimate position of the prosthesis in vivo (H1 hypothesis of equivalence).

## Materials and methods

Prior to this study, the operating surgeon had experience with over 200 TKA performed using PSPG. For this study, 26 patients were included. Inclusion criteria were: painful and disabled knee joint resulting from osteoarthritis, ability and willingness to follow instructions, including control of weight and activity level.

Exclusion criteria were: failure of previous joint replacement; pregnancy; previous knee surgery, except for arthroscopic meniscectomy; metal near knee joint (MRI scan not possible); not able or willing to undergo MRI scan and CT scan.

The cohort consisted of 13 women and 13 men with an average age of 66 (range 52–83 years). All eligible patients were approached to participate in this study from June 2011 and onwards. Patients received oral and written information, and when informed consent was signed, patients were included in this study. The first 26 consecutive patients that gave informed consent were included. Last patient was included December 2011.

Pre-operative MRI scanning of the hip, knee and ankle was performed 6 weeks prior to surgery according to the standard Signature scanning protocol. Software (Mimics, Materialise NV, Leuven, Belgium) was used to create virtual three-dimensional models of femur and tibia. The program was used to determine appropriate implant size and positioning of the knee prosthesis (Vanguard™ Complete Knee System, Biomet, Inc., Warsaw, IN) for each patient individually. For the purpose of this study, an additional full-leg CT scan of the ipsilateral leg was performed pre-operatively (radiation dose for single CT scan: 5.69 mSv = equivalent to half the dose of a CT pelvis or CT thorax). This scan was made according to a standardised scanning protocol.

A digital, virtual plan of the proposed peroperative positioning was sent to the surgeon. The surgeon was able to adjust the digital plan when deemed necessary. After approving the digital plan, guides for peroperative use were manufactured using a rapid prototype engineering technique. Operative procedure using these guides was described in detail by Boonen et al. [4]. Intraoperatively, the practical form and fit of the guides and all peroperative changes from the pre-operative plan (level of resection, size of prosthesis) were registered.

Six weeks post-operative, the full-leg CT scan of the operated extremity was scheduled according to the same standardised scanning protocol as to which the pre-operative CT scan was made. In order to define the difference between the pre-operative digital planning and post-operatively achieved alignment results, the post-operative CT scan should need to be compared with the digital plan based on the pre-operative MRI scan. However, direct comparison between this pre-operative MRI scan and the post-operative CT scan is difficult and inaccurate as the MRI scan is a local knee scan, and matching would therefore be difficult post-operatively as a great deal of referencing points have disappeared with prosthesis placement.

**Fig. 1:**
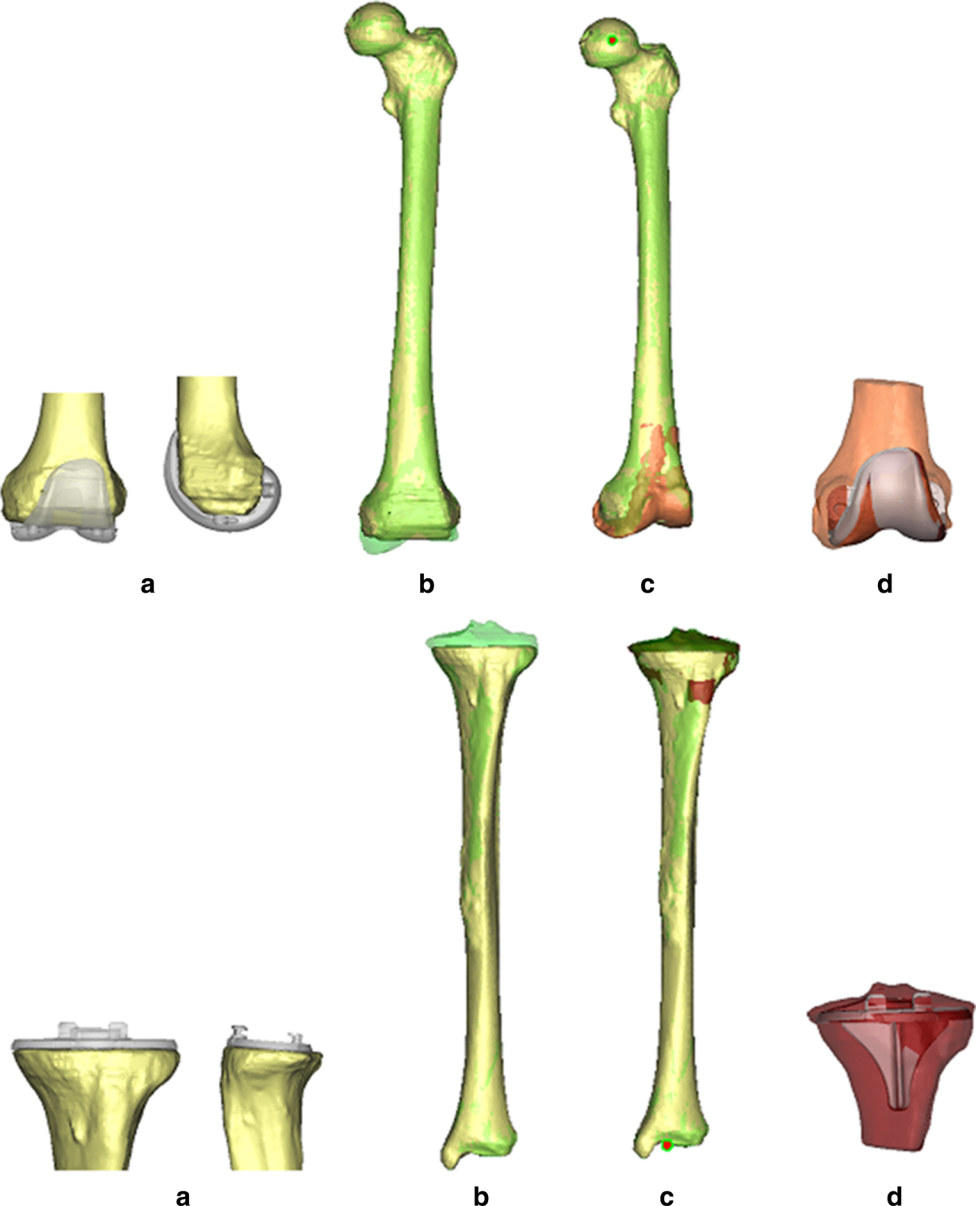
Registration process for the femur (*upper row*) and tibia (*lower row*). **a** Implant STL registered on post-op femur (*yellow*). **b** Post-op femur (*yellow*) on pre-op femur (*green transparent*). **c** CT femurs registered on MRI femur (*red transparent*) and plan (*hip point*, *red*). **d** Pre-op planned implant (*red*) with post-op implant (*grey transparent*) position

**Fig. 2:**
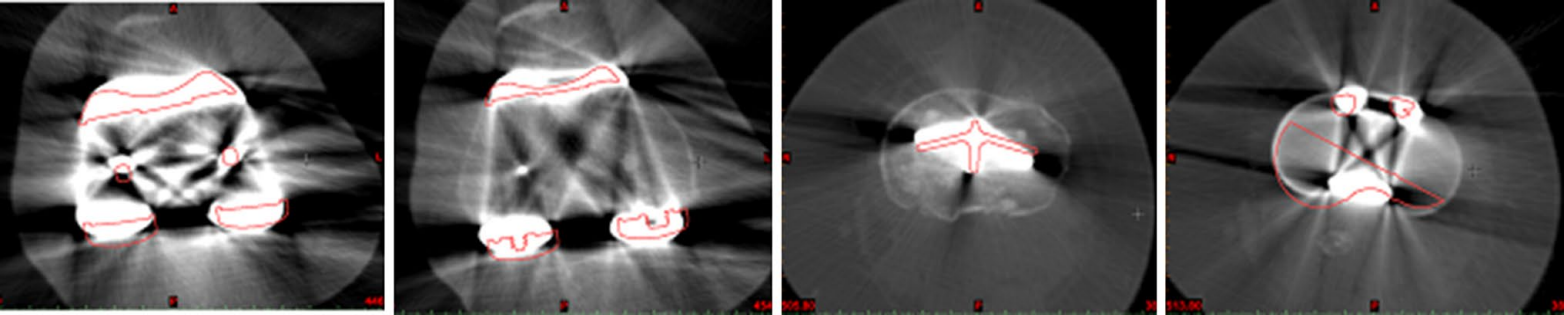
Example of the post-op CT scan images of femur (*first two images*) and tibia (*last two images*) with pre-op plan superimposed (*red*). This registration permits measurement of rotational alignment in the transverse plane

To resolve this problem, the pre-operative full-leg CT scan was made next to the pre-operative MRI scan to serve as an intermediate step in the registration process. The MR images could be matched to the pre-operative CT images as digital 3D models of both scans were generated for the femur and tibia using the 3-matic software of Materialise NV (Materialise NV, Leuven, Belgium). Advantage of this way of 3D registration is that it makes the results independent of scan orientation and leg position during scanning. After surgery, 3D reconstruction femur and tibia models of the post-operative CT scan could be superimposed onto the pre-operative CT models that represented pre-planned cuts and prosthesis placement. In this way, exact comparison could be performed between pre-operatively planned resections and prosthesis placement and ultimate resections and placement in vivo (Fig. 1). Measurements taken using this technique have been reported to be substantially more accurate compared with conventional radiographs and 2D CT scans with intra-observer reliability (ICC) ranging from 0.73 to 0.99 and inter-observer reliability (ICC) ranging from 0.89 to 0.99 [13]. Deviations (in degrees) from pre-op planning in all three anatomical planes for femoral and tibial component were determined (Fig. 2). Positive values indicate varus, flexion/posterior slope, exorotation, and negative values indicate valgus, extension/anterior slope, endorotation deviations relative to the pre-operatively calculated position. Outliers (defined as deviations more than 3° from pre-operatively planned position) were calculated in each plane and for the individual prosthesis components. Accuracy of measurements was to within 0.1 degree.

The local ethics committee approved this prospective cohort study (institutional review board Atrium–Orbis–Zuyd, number: 11-T-15, date: 2 March 2011).

### Statistical analysis

For this study, two one-sided tests (equivalence test) were used to obtain the sample size (H1: mean of difference = 0). According to several studies, in the frontal plane a post-operative range for alignment of the mechanical axis of the leg of maximal 3° of valgus to 3° of varus is acceptable. Standard deviation of difference was estimated to be 5. This was based on our pilot study in which we found a range of 7° of varus to 5.4° of valgus [12]. Significance level was determined at 0.05 (alfa), and power was set at 80 %. According to this calculation, 26 patients should be included in this prospective cohort study.

Two one-sided tests (TOSTs) will be performed to examine whether the null hypothesis (H0: the difference of pre-operative planning and post-operative alignment is more than +3 or −3) can be rejected. The margin is specified for either side, and both one-sided tests have to be rejected to establish equivalence.

## Results

Mean pre-operative mechanical axis was 0.9° varus (range: 15.3° varus–10.1° valgus). Eighteen patients had varus knee osteoarthritis and eight patients valgus knee osteoarthritis.

### 3D CT scan analysis

For the femoral component in all three planes and for the tibial component in frontal and sagittal planes, there was no statistically significant difference between pre-operative planning and achieved position post-operatively in either plane, because the two one-sided tests were rejected and the alternative hypothesis of equivalence was accepted. For the tibial component in transverse plane, the null hypothesis of a priori specified difference was not rejected and equivalence could therefore not be established. Results of the measurements for the femoral component are summarised in Table 1 and for the tibial component in Table 2.

**Table 1:** Measurements for femoral component

	Frontal plane	Sagittal plane	Transverse plane
Mean absolute deviation from pre-op planning (SD)	1.8° (1.3)	2.5° (1.6)	1.6° (1.4)
Mean deviation from pre-op planning +3	−3.6	−4.0	−3.2
Mean deviation from pre-op planning −3	2.4	2.0	2.8
95 % CI deviation from +3	−4.5; −2.8	−5.1; −2.8	−4.1; −2.4
95 % CI deviation from −3	1.5; 3.3	.89; 3.2	1.9; 3.7
Range	−5°; 4°	−6°; 5°	−6°; 3°
% Outliers >3°	7.7	19.2	3.8
*P* value equivalence testing*			
Deviation >+3	<0.001	<0.001	<0.001
Deviation <−3	<0.001	0.001	<0.001

* *P* value for equivalence tests with two one-sided tests (TOSTs). If both tests are rejected, the non-equivalence hypothesis is rejected, and the alternative hypothesis is concluded at the 0.05 significance level

**Table 2:** Measurements for tibial component

	Frontal plane	Sagittal plane	Transverse plane
Mean absolute deviation from pre-op planning (SD)	1.7° (1.2)	1.7° (1.5)	3.2° (3.6)
Mean deviation from pre-op planning +3	−2.1	−3.2	−1.1
Mean deviation from pre-op planning −3	3.9	2.8	4.9
95 % CI deviation from +3	−2.8; −1.3	−4.1; −2.2	−2.9; .69
95 % CI deviation from −3	3.2; 4.7	1.9; 3.8	3.2; 6.7
Range	−3°; 5°	−5°; 5°	−5°; 16°
% Outliers >3°	3.8	7.7	23.1
*P* value equivalence testing*			
Deviation >+3	<0.001	<0.001	0.222
Deviation <−3	<0.001	<0.001	<0.001

* *P* value for equivalence tests with two one-sided tests (TOSTs). If both tests are rejected, the non-equivalence hypothesis is rejected, and the alternative hypothesis is concluded at the 0.05 significance level

The absolute mean deviation from planned mechanical axis was 1.9° (range: −4° to 7°), the two one-sided tests were rejected, and the alternative hypothesis of equivalence was accepted. 11.5 % of the values were above the threshold set as outlier for the mechanical axis.

**Fig. 3:**
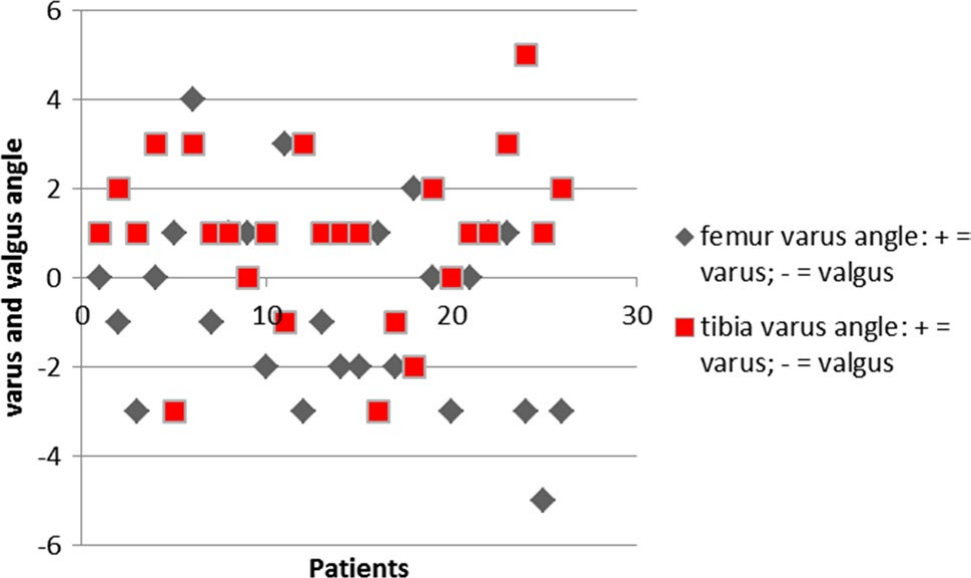
*Scatter plot* in which the deviations in degrees (*Y* axis) of the femoral (in *grey*) and tibial (in *red*) components form intended position (pre-op plan) are presented for all patients individually (*X* axis) in the frontal plane (varus as positive values; valgus as negative values)

**Fig. 4:**
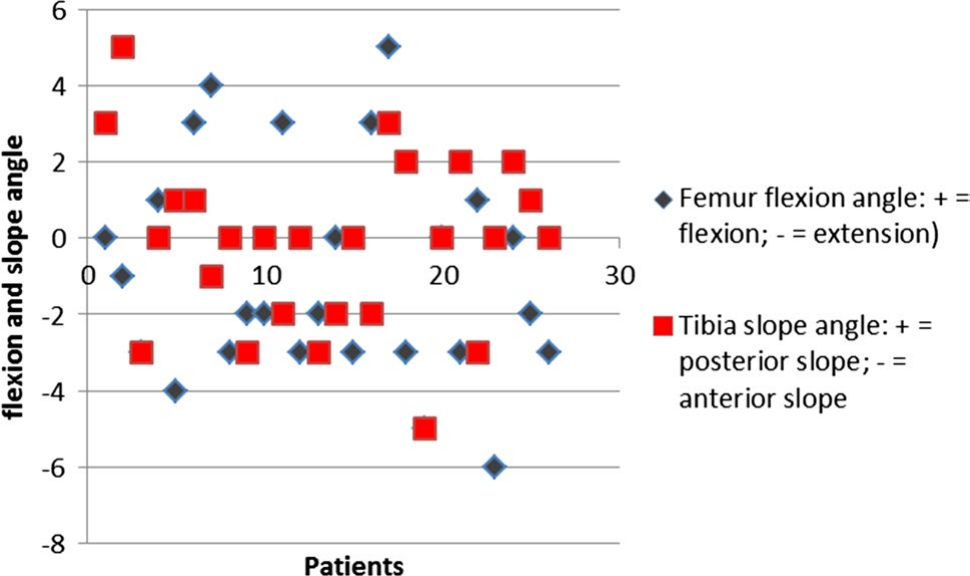
*Scatter plot* in which the deviations in degrees (*Y* axis) of the femoral (in *grey*) and tibial (in *red*) components form intended position (pre-op plan) are presented for all patients individually (*X* axis) in the sagittal plane (flexion and posterior slope as positive values; extension and anterior slope as negative values)

**Fig. 5:**
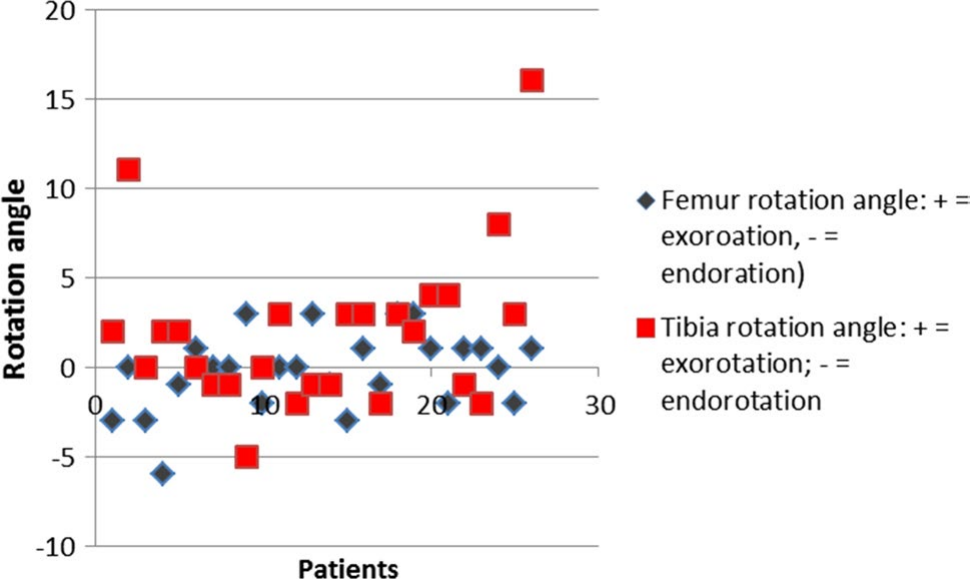
*Scatter plot* in which the deviations in degrees (*Y* axis) of the femoral (in *grey*) and tibial (in *red*) components form intended position (pre-op plan) are presented for all patients individually (*X* axis) in the transverse plane (exorotation as positive values; endorotation as negative values)

**Fig. 6:**
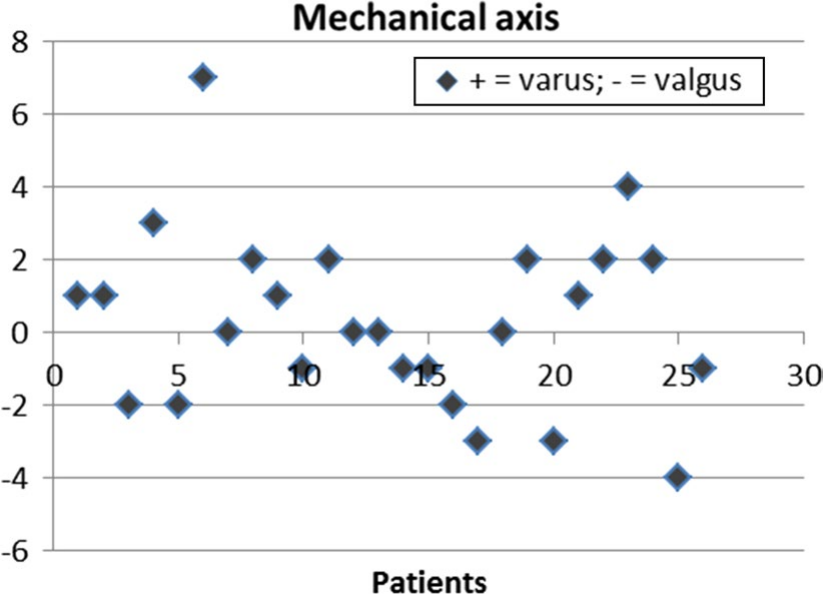
*Scatter plot* in which the deviations from the intended neutral mechanical axis are presented for all patients individually. Positive values indicate varus mismatch, and negative values indicate valgus mismatch

Percentages of outliers more than 3° from intended position (pre-op plan) for the femoral component were 7.7, 19.2 and 3.8 in the frontal, sagittal and transverse planes, respectively. Percentages of outliers more than 3° from intended position (pre-op plan) for the tibial component were 3.8, 7.7 and 23.1 in the frontal, sagittal and transverse planes, respectively. Figures 3, 4 and 5 show scatter plots in which the deviations of the femoral and tibial components form intended position (pre-op plan) are presented for all patients individually in the frontal, sagittal and transverse planes, respectively. Figure 6 shows a scatter plot in which the deviations from the intended neutral mechanical axis are presented for all patients individually.

### Operative data

All guides fitted well on the native anatomy of the individual patients, and no conversions to traditional alignment techniques were necessary. Correct fit was defined as a stable fixation of the guides on the native bone and cartilage and the absence of mismatch between the contours of the cartilage/bone and the contours of the guides. During surgery, in one patient, an extra 2 mm had to be resected from the distal femur, and in three patients, an additional 2 mm was resected from the tibia. The sizes of four femoral components were adjusted one size (two cases downsized and two cases upsized), and four tibial components were adjusted one size (two cases downsized and two cases upsized) during surgery to obtain a better fit of the components.

**Fig. 7:**
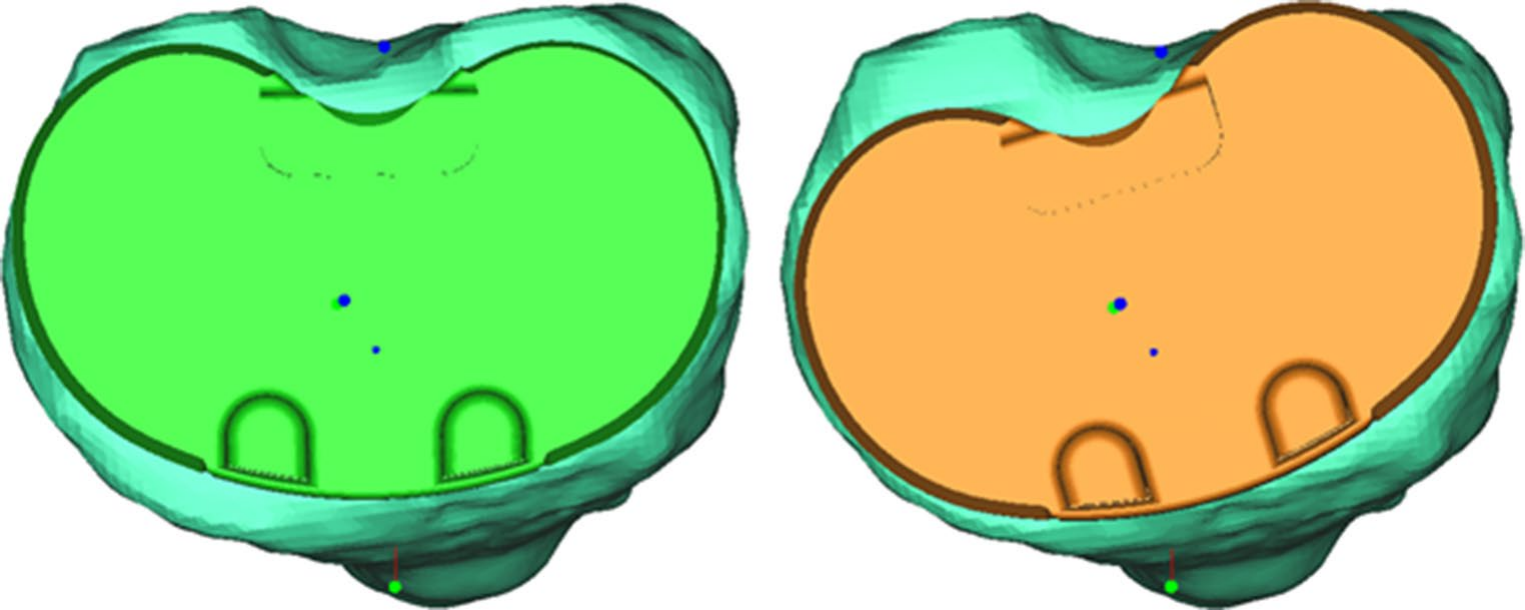
Example of how the contours of the proximal tibia are guiding when drill holes that dictate rotation cannot be retrieved. On the left the position of tibial plateau (*green*) as calculated pre-operatively. On the right the position of tibial plateau (*orange*) as detected post-operatively

## Discussion

The most important finding of the present study was that there was no statistically significant difference between pre-operative planning and achieved position post-operatively in either plane, except for the tibial component in the transverse plane.

Mean deviations from pre-op planning were all within 3°, except for the tibial component in the transverse plane, in which mean absolute deviation from pre-op planning was 3.2°. The ranges of deviations were small in all planes (maximal deviation from planning 6°), with the exception of tibial rotation in which the maximal deviation was 16°. Furthermore, the technique showed a reliable restoring of a neutral mechanical axis in accordance with the pre-operative plan. Percentages of outliers are small in all planes (ranging from 3.8 to 7.7 % outliers) except for the femoral component in the sagittal plane (19.2 % outliers) and for the tibial component in the transverse plane (23.1 % outliers). When comparing our results to the percentages of outliers with conventional instrumentation (CI) and computer-assisted surgery (CAS) in recent literature, our results are comparable to results obtained with CAS (Table 3).

**Table 3:** Comparison of PSPG results in our study with results of CI and CAS in recent literature

	PSPG (%)	Conventional (%)	CAS (%)
Mechanical axis	11.5	26.9 and 28.3*	9.5 and 12.2*
Femur frontal plane	7.7	15.8–16.2*	4.7–5.1*
Tibia frontal plane	3.8	8.6–11.6*	4.0–4.2*
Femur sagittal plane	19.2	36.6–41.3*	18.6–19.8*
Tibia sagittal plane	7.7	21.8–31.6*	13.6–23.2*
Femur transverse plane	3.8	14.8^†^	17.1^†^
Tibia transverse plane	23.1	33.3^†^	32.7^†^

Comparison of percentages of outliers obtained with PSPG in our study compared to conventional instrumentation and computer-assisted surgery (CAS)

* According to Thienpont et al. [26] and Cheng et al. [7]

^†^According to Cheng et al. [7]

There are several steps in the process of guide fabrication to prosthesis alignment that are potential sources of error. In sequential order, these include: imaging (MRI or CT scan), landmark registration, calculation algorithm for constructing the digital plan, rapid prototyping production process of guides, peroperative fit of the guides, peroperative handling of the guides, sawing/bone cuts, cementing technique and position of the knee during cement hardening. In this study, the combined potential errors from the production process up to and including the position of the knee during cement hardening ware assessed.

Relatively higher percentages of outliers for the femoral component in the sagittal plane and for the tibial component in the transverse plane were observed. Several explanations can be given for the observed outliers in these planes. The inaccuracies in the sagittal plane for the femoral component are believed to be caused mainly by the microplasty instrumentation that was used in this study. The Signature guides are designed to guide positioning of traditional cutting blocks and have no sawing sleeves incorporated into their design. Therefore, for making the chamfer cuts, the traditional sliding instrument was used. This instrument, that is also frequently used with conventional intramedullary alignment of the Vanguard knee system (Biomet inc., Warsaw, IN, USA), can sometimes not be fixed stably on the surface of the distal femoral bone resection. We believe therefore that special attention should be given to users of microplasty instrumentation, a sliding four-in-one cutting block for the femur, as the use of these instruments might predispose to outliers in the sagittal plane. In addition, it might be that part of the outliers in this plane was caused by the sliding of the femoral guide into flexion or extension when drilling the guide into place.

As for the rotational alignment of the tibia in the transverse plane, percentages of outliers were higher than expected with three cases (11.5 %) in which rotation deviated more than 5° from planning. There are several possible explanations for this. First, the tibial guide has a tendency to slide laterally when positioned on the tibia. This might lead to tibial component placed slightly more in external rotation. Secondly, especially in osteoporotic bone, the proximally drilled pin holes (that dictate rotation) can be difficult to retrieve after having performed the horizontal cut for the tibia. In that case, there is a tendency to follow the contours of the resected proximal tibia when positioning the guiding instrument for the tibial punch, resulting in preparing the proximal tibia in such a way that relative exorotation of the component arises (Fig. 7).

Smaller deviations from planning in the frontal and sagittal planes could also be the result of the sawing itself. Bäthis et al. [2] analysed the resection process using an accurate CT-based navigation system and found deviations related to the sawing process between 0.5° and 1.0°. These cutting errors were independent of the surgeon’s experience [17]. Besides the above-mentioned inaccuracies resulting from the sawing itself, Catani et al. [5] observed that cementation and impaction of the final components can introduce a considerable error (up to 3° in the sagittal plane for the tibia) in alignment, regardless of how accurately the resection planes were made.

Given these observations, it is clear that there are some potential pitfalls that might occur during surgery itself when using PSPG that could jeopardise the guide potential for achieving adequate component alignment. We believe that, when positioning of the tibial guide, it is important to aim at pressing the guide on the medial part of the tibia and thus avoiding lateralisation (and with that also external rotation) of the guide. Additionally, before positioning the guiding instrument for the tibial punch, the holes in the tibial plateau that determine rotation should be visible. It is advisable to use the pulse lavage to make these pinholes better visible. Furthermore, we advise against using the sliding version from the four-in-one cutting block for preparation of the femur, and we stress the importance of a stable fixation of the guide on the femur before drilling it in place. Furthermore, the PSPG system analysed in this study is a bone referencing technique, meaning that proper ligament balancing after bony resections is absolutely mandatory.

Several authors have published their results on the alignment obtained with PSPG. Diverse systems have been subject of study, and results concerning outliers in alignment with PSPG differ greatly. Higher-quality studies report both superior results from PSPG with respect to the percentage of outliers of the individual components when compared to conventional instrumentation [9, 25] as comparable results [1, 3, 6, 18, 22, 27]. These studies, however, use standard radiographs, long-leg radiographs or 2D CT scans to assess alignment. This is substantially less accurate than 3D CT scans, which were used in this study [13]. In addition, other studies use reference axes and reference points on post-operative imaging that are not all equal to the reference points and axes used to calculate implant position and subsequently resulting in the pre-operative digital plan. This, as already stated, makes direct comparison between post-surgery alignment and proposed alignment in the digital plane difficult. Computer navigation has been used in some studies in order to overcome these limitations. Three of four studies [8, 15, 23] report a higher percentage of outliers (more than 3° deviation from intended bone cuts) than the percentages reported in our study. The fourth study, also investigating the Signature system, reports comparable percentages of outliers in the investigated frontal and sagittal planes [24]. Limitation of using navigation to assess guide accuracy is the inherent error with respect to landmark registration [14], a limitation that was overcome with the use of our study design. Additionally, data in this study were not influenced by the position of the leg during scanning, as could be a source of error in other studies.

There are weaknesses in this study. Clinical outcomes were not taken into account since the study was not adequately powered to make valid conclusions on clinical outcome. The purpose of this study was to compare final alignment outcome with the pre-operative plan, and assembling a control group was therefore not applicable (no pre-operative digital plan in control group). We therefore chose to compare our results with the literature, however, for the purpose of framing only.

Given the conflicting results on alignment with PSPG in the literature and given the mentioned potential pitfalls when using PSPG highlighted in this study, we believe that PSPG cannot automatically be seen as a technique that enables the less experienced knee surgeons to obtain optimal alignment results. We think that there are still numerous crucial steps in order to achieve optimal alignment results when using PSPG systems. Therefore, we recommend adequate training surgeons before starting using PSPG in a day-by-day practise.

## Conclusion

The results of this study indicate that overall PSPG is a reliable technique for aligning the components of a TKA and for adequately restoring a neutral mechanical axis. The observed inaccuracies, mainly in rotational alignment for the tibia, are explained by the cutting and prosthesis placement but mainly illustrate possible pitfalls with this technique. These potential pitfalls need attention and highlight the need for adequate surgeon training and guidance when starting with PSPG.
